# Remarks on the Condition of Apothecaries, with Suggestions for a New Bill

**Published:** 1814-03

**Authors:** 


*For the Medical and Physical Journal.*

### *Remarks on the Condition of Apothecaries, with Suggestions for a new Bill.*

IT is a notorious fact, and must be acknowledged by all classes of society, that a reform in the profession of the apothecary is absolutely necessary, not only for the encouragement of qualified practitioners, but more particularly for the benefit that would arise to the army, navy, and country in general, from a judicious arrangement, under the sanction and protection of government.

We cannot expect that privileges will be taken away from certain professions or descriptions of people, and given to us. On the other hand, we ought not voluntarily to make such great and serious sacrifices, without a prospect of some certain advantage to be derived from it; as would be the case were the present amended bill to be passed into a law.

If apothecaries were allowed by it to make demands for personal attendance, which might be limited not to exceed half the ordinary fees of physicians for similar attendance, they might and ought then to charge less for medicines prepared. The consequence would be, that all prescriptions would invariably be brought to the apothecaries, and the profit profit to them, in this respect alone, would undoubtedly be greater than at present, and the fees would then be an extra profit; whereas, according to the amended bill, nothing can prevent the druggist from reaping, at least, one half the emolument that properly belongs to the apothecary, and which will be irrecoverably lost if it should be passed into a law.

Impressed with this opinion, I have not the least doubt that it would become a heavy burthen upon us which we could never remove. I should, therefore, recommend that unless the apothecaries be permitted to make demands for attendance, the present bill be withdrawn. I know of no profession, except ours, which is excluded the natural privilege of demanding payment for trouble, attendance, time, or whatever name may be given to it; so that, in its present state, there is no inducement whatever for men of liberal education to enter into the profession.

The following, as the basis of a bill, I conceive, though imperfect, would be extremely beneficial to the apothecary, as well as to society at large.

1st, That a medical board be established by government for the examination of medical practitioners, to be called apothecaries, who will be then entitled to practise as such in the army, navy, or as private practitioners.

2dly, That no person whatsoever be allowed to practise under the name of an apothecary, alone, or conjointly with any other profession, unless he has actually served a regular apprenticeship to an apothecary, or can produce testimonials of having attended as a pupil of a public hospital, and a regular course of medical lectures.

3dly, That none be permitted to commence business hereafter, as an apothecary, (surgeons in the army and navy excepted,) without being found duly qualified, on examination, by the medical board.

4thly, That the indenture of every apprentice, in future, bear a stamp of 25*l.*

5thly, That apothecaries, whether combined or not with other professions, be allowed to demand fees for attendance, visits, or journies, not exceeding one half the ordinary fees of physicians in general for similar duties.

If this plan were approved, it would be adviseable to petition his Royal Highness the Commander in Chief, and the Board of Admiralty, for their approbation and interest, explaining to them the advantages likely to result from the respectability and increased number of qualified men, &c.

